# Taxonomic contribution to the *Aleiodes melanopterus* (Erichson) species-group (Hymenoptera, Braconidae, Rogadinae) from Brazil

**DOI:** 10.3897/zookeys.142.1705

**Published:** 2011-10-31

**Authors:** Eduardo Mitio Shimbori, Angélica Maria Penteado-Dias

**Affiliations:** 1 Embrapa Agropecuária Oeste, BR 163, km 253.6, Dourados, MS, Brazil; 2 Universidade Federal de São Carlos, Rodovia Washington Luiz, km 235, CEP 13 565-905, São Carlos, SP, Brazil

**Keywords:** Brazil, new species, *Eucystomastax*, distribution

## Abstract

The *Aleiodes melanopterus* (Erichson, 1848) species-group includes 21 species, of which seven are known from the Neotropical region: *Aleiodes flavistigma* Shaw, 1993, *Aleiodes lucidus* (Szépligeti, 1906), *Aleiodes melanopterus*, *Aleiodes mexicanus* Cresson, 1869, *Aleiodes politiceps* (Gahan, 1917), and the new species *Aleiodes shaworum* **sp. n.** and *Aleiodes vassununga* **sp. n.** Distribution ranges of *Aleiodes melanopterus*, *Aleoides flavistigma* and *Aleiodes lucidus* are extended and the female of *Aleiodes lucidus* is described. A key to the Neotropical species of this species-group is presented.

## Introduction

The Neotropical species of *Aleiodes melanopterus* (Erichson, 1848) species-group has been treated as several different genera until Shaw (1993) clarified its relationship with others *Aleiodes* species-groups. The Neotropical lineage was then proposed to be a subgenus within *Aleiodes*, namely *Eucystomastax* Brues, 1912, including five nominal species: *Aleiodes flavistigma* Shaw 1993, *Aleiodes lucidus* (Szépligeti 1906), *Aleiodes melanopterus* (Erichson, 1848), *Aleiodes mexicanus* Cresson 1869 and *Aleiodes politiceps* (Gahan, 1917) (Yu et al. 2005). In a broad sense the *Aleiodes melanopterus* group is present in Palaearctic, Nearctic, and Neotropical regions (Marsh and Shaw 1999), and includes 21 species, composing a monophyletic group defined by the large oral space and narrow clypeus (Fortier and Shaw 1999). All Neotropical species plus *Aleiodes politiceps* (Gahan, 1917) comprises a derived subgroup within the *Aleiodes melanopterus* group, defined by having pectinate tarsal claws and strongly protruding clypeal carina (Shaw 1993; Fortier and Shaw 1999).

## Material and methods

A portion of examined specimens, deposited at DCBU (Universidade Federal de São Carlos), comes from several different surveys throughout Brazil. Additional specimens were loaned from several entomological collections in Brazil, deposited temporally at DCBU: Museu Paraense Emilio Goeldi (MPEG), Instituto Nacional de Pesquisas da Amazônia (INPA), Museu Nacional do Rio de Janeiro (MNRJ), Museu de Zoologia da Universidade de São Paulo (MZUSP), Coleção Entomológica Padre Jesus S. Moure – Departamento de Zoologia da Universidade Federal do Paraná (DZUP). We examined 194 specimens of the *Aleiodes melanopterus* species-group, all collected in Brazil.

Terminology used mostly follows Shaw (1993), exception made for some microsculpture characters that follows Marsh & Shaw (1999). Abbreviations used in the descriptions follows strictly that used by Shaw (1993), namely: BL= body length, excluding antenna and ovipositor; FWL= fore wing length; F#= flagellum #; MS= malar space; EH= maximum eye height; EW= maximum eye width; TW= temple width; OS= oral space, maximum width in anterior view; OOD= ocellar–ocular distance (shortest distance from eye margin to lateral ocellus); OD= ocellus diameter (maximum width of lateral ocellus); T#= tergum #; OL= ovipositor length; HBTL= hind basitarsus length; HTS= hind tibial spur length (longest spur); R#= radius segment #. Colour pictures were taken by stereomicroscope. Greyscale pictures were taken at SEM in low vacuum.

## Results

We identified all previously known South American species among examined material, i.e. *Aleiodes flavistigma* Shaw 1993*, Aleiodes lucidus* (Szépligeti 1906)*, Aleiodes melanopterus* (Erichson, 1848); plus two new species described bellow: *Aleiodes shaworum* sp. n. and *Aleiodes vassununga* sp. n. One of the specimens examined constitutes the first recorded female of *Aleiodes lucidus*, and also the first record of this species from Brazil. The distribution range of *Aleiodes flavistigma* is extended to Minas Gerais State in Brazil; this is the first record of this species outside of Santa Catarina State. Some morphological features of the Brazilian *Aleiodes melanopterus* specimens are described and discussed.

### Key to Neotropical species of Aleiodes melanopterus (Eucystomastax) species-group (modified from Shaw 1993)

**Table:** 

1	Metasoma orange to reddish brown (Fig. 2)	2
–	Metasoma black apically or mostly black (Fig. 1)	4
2	First and second metasomal terga striate; body bicoloured, head and legs black; malar space narrow, about ½ basal width of mandible	*Aleiodes mexicanus* Cresson
–	First and second metasomal terga strongly costate (Fig. 14); body unicoloured (Fig. 2); malar space about equal to basal width of mandible	3
3	Pterostigma yellow (Fig. 2)	*Aleiodes vassununga* sp. n.
–	Pterostigma black	*Aleiodes politiceps* (Gahan)
4	Notauli absent or very shallow anteriorly, mesonotum entirely smooth (Fig. 10); epicnemial carina effaced dorsally or completely absent (Fig. 11)	*Aleiodes lucidus* (Szépligeti)
–	Notauli distinct, although smooth (Fig. 8); epicnemial carina entirely present (Fig. 12)	5
5	Pterostigma yellow	*Aleiodes flavistigma* Shaw
–	Pterostigma black	6
6	Clypeus strongly protruding (Fig. 7); hind coxa smooth dorsally; mesonotum orange, pronotum and fore coxa often orange	*Aleiodes melanopterus* (Erichson)
–	Clypeus not strongly protruding (Fig. 6); hind coxa striated dorsally (Fig. 9); pronotum, mesonotum and fore coxa black (Fig. 1)	*Aleiodes shaworum* sp. n.

#### Aleiodes flavistigma

Shaw, 1993

http://species-id.net/wiki/Aleiodes_flavistigma

##### Material examined.

Brazil: 3 females, Nova Teutônia, SC, X.1967, F. Plaumann col.; 1 female, Extrema, MG, 25.XII.1990, E. Mariano col.

##### Distribution.

Brazil, Minas Gerais and Santa Catarina States.

#### Aleiodes lucidus

(Szépligeti, 1906)

http://species-id.net/wiki/Aleiodes_lucidus

Figs 10, 11

Macrostomion lucidus Szépligeti 1906: 609

##### Material.

Female. Fazenda São João, Diamantina, MT, Brazil, 450m, 5.II.1981, Ekis & Young col.

##### Description.

Female. Body length 12 mm, fore wing length 11.6 mm.

Head. Flagellum broken at F40, F1 2 times longer than wide, F2 1.8 times longer than wide, flagellomeres beyond second about as long as wide; malar space ¾ basal width of mandible; MS/EH 0.33; TW/EW 0.67; occipital carina weak ventrally, not meeting the hypostomal carina; oral opening width 1.16 times clypeo–antennal distance; clypeus height 0.3 times its width, protruding and bordered by carina; OS/MS 2.08; OOD/OD 1.33; face smooth, swollen medially; temple smooth; maxillary palpal segments 2–4 swollen.

Mesosoma. Entirely smooth; notauli very shallow anteriorly, otherwise absent (Fig. 10); epicnemial carina nearly absent (Fig. 11); precoxal sulcus absent; propodeum with median carina complete. Legs:tarsal claws pectinate; hind coxa smooth dorsally.Wings: dusky; R1/R2 0.42; R1/recurrent vein 0.62; 1CUa/1cu-a 1.85; basella/mediella 0.4.

Metasoma. T1 length/width 1.15; T2 length/width 0.7; T3 length/width 0.58; all terga smooth except for medio-longitudinal carina on T1 and T2; OL/HBTL 0.22; HTS/HBTL 0.3.

Colour. Body colour black, except mesothorax orange to infuscate orange.

##### Distribution. 

Bolivia, Mapiri and Santa Cruz; Brazil, Mato Grosso.

##### Comments.

This species was known only from male until the present study. The female is very similar to male, but has a larger body size, and differs in some wing vein proportions and the smoother face. The diagnostic characters of the species (e.g. notauli virtually absent, whole body with smooth sculpturing, reduced epicnemial carina) are present in the female specimen examined.

#### Aleiodes melanopterus

(Erichson, 1848)

http://species-id.net/wiki/Aleiodes_melanopterus

Figs 3, 7

Rogas melanopterus Erichson 1848: 588
Macrostomion peruvianum Szépligeti 1904: 193
Rhogas rufithorax Cameron 1911: 313
Rhogas fortipalpus Cameron 1911: 314
Rhogas forticarinatus Cameron 1911: 314
Eucystomastax bicolor Brues 1912: 223

##### Material examined.

74 females and 108 males. Brazil. Acre (AC): Cruzeiro do Sul, Rio Branco; Amazonas (AM): Serra dos Porcos; Espírito Santo (ES): Fundão, Santa Teresa; Goiás (GO): Anápolis, Goiânia – “Campinas”, Jataí; Mato Grosso (MT): Aripuanã, Alta Floresta, Barra do Tapirapé, Chapada dos Guimarães, Itiquira, Jaçanã – P.N. Xingu, Rondonópolis; Mato Grosso do Sul (MS): Campo Grande, Dourados; Minas Gerais (MG): Araxá, Corinto, Passos; Pará (PA): Barreirinha, Belém, Canindé, Parauapebas – Serra Norte, Peixe-Boi, Redenção – Gorotire, São Félix do Xingu, Serra Norte; Paraná (PR): Curitiba, Maringá, Ponta Grossa, Rolândia, São José dos Pinhais; Rio de Janeiro (RJ): Santa Maria Madalena; Rio Grande do Sul (RS): Santa Maria, São Leopoldo; Rondônia (RO): Ouro Preto d'oeste, Porto Velho, Vilhena; Santa Catarina (SC): Nova Teutônia; São Paulo (SP): Barueri, Cananéia – Ilha do Cardoso, Castilho, Caraguatatuba, Juquitiba, Luís Antônio, Monte Alegre, Nova Europa, Onda Verde, Rio Claro, Salesópolis, São Carlos, São Paulo, Tabatinga, Ubatuba, Vargem Grande Paulista.

##### Morphological notes on Brazilian specimens.

Head. Occipital carina of all examined specimens is absent ventrally, thus occipital and hypostomal carina do not meet (Fig. 7). This is probably a misinterpreted character in Shaw (1993) corrected by Fortier and Shaw (1999) (character 20, state 1 for *melanopterus*); face sculpture variable, smooth to rugose, rugosity concentrated near raised median area when present (Fig. 3).

Colour variation. The examined specimens present distinct colour pattern variation. Propodeum always orange; metasoma black in virtually all specimens (97%); hind coxa black (99% of specimens); mid coxa somewhat lighter than hind; fore coxa bright orange in 97% of the specimens, contrasting with remainder black leg; one of the examined specimens has dark pronotum and propleuron.

##### Distribution.

South America, from Suriname to Northern Argentina (North to South), and from South-eastern Brazil to Eastern Peru (East to West). Not recorded in the East of Andean Cordillera, Central America (Shaw 1993), and Northeast Brazilian region. South American countries with records: Argentina, Bolivia, Brazil, Ecuador, Paraguay, Peru and Suriname.

#### Aleiodes shaworum sp. n.

urn:lsid:zoobank.org:act:27156FC8-61DA-4E1C-9065-5B68F29B3522

http://species-id.net/wiki/Aleiodes_shaworum

**Figures 1, 2:**
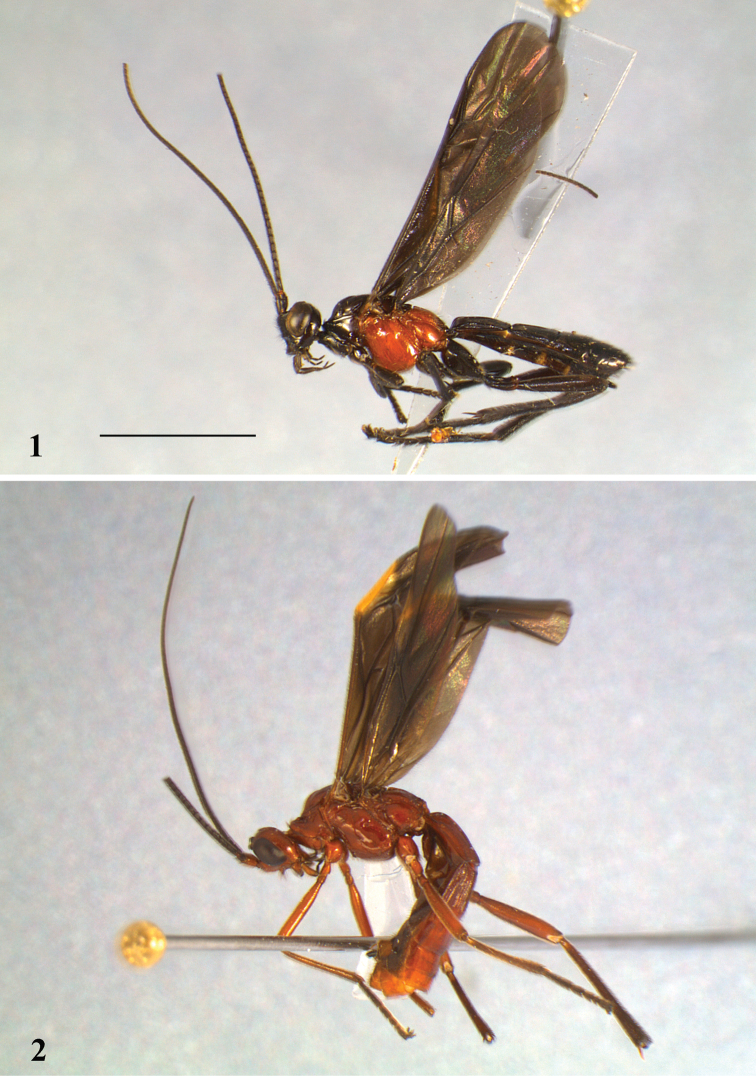
(scale line = 4 mm). Habitus left: **1** *Aleiodes shaworum* sp. n. **2** *Aleiodes vassununga* sp. n.

Figs 1 4, 6, 8 9, 12, 13 15

##### Material.

Type locality: Brazil, São Paulo, Juquitiba, Sítio Sonho do Vovô, Atlantic forest.

Type specimens. Holotype, female, pinned. Original label: “Sítio Sonho do Vovô, Juquitiba – SP – 22-IV-1988 – (V) L.A. Joaquim, col.” DCBU / UFScar, São Carlos.

Paratypes (DCBU): 1 female, same as holotype; 2 males, Brazil, Barueri, SP, 11.V.1966 and 22.I.1966; 1 male: Brazil, Estação Florestal, Caraguatatuba, SP, 40 m, VII.1965.

##### Diagnosis.

This species is similar to *Aleiodes melanopterus*. It can be distinguished by colour pattern: mesonotum black, contrasting with orange mesopleuron, metapleuron, and propodeum; smaller oral opening and clypeus not strongly protruding; striated sculpture on dorsal part of hind coxa; fore wing vein R1 relatively shorter.

##### Description.

Female. Body length 9–9.2 mm, fore wing length 8.2 mm.

Head (Fig. 4). Flagellum with 60 flagellomeres, F1 2.5–3 times longer than wide, F2 about twice longer than wide, remaining flagellomeres almost twice as long as wide; malar space ½ basal width of mandible; MS/EH 0.2; TW/EW 0.62; occipital carina absent ventrally, not meeting hypostomal carina (Fig. 6); oral opening width slightly greater than clypeo–antennal distance; clypeus height/width 1/3; clypeus not protruding, without carinate boarder; OS/MS 3.1–3.2; OOD/OD 0.87; face rugo-striate; frons and vertex smooth; temples smooth scattered with punctuations near occipital carina; maxillary palpus swollen, especially segments 2 and 3.

Mesosoma(Fig. 8). Pronotum smooth laterally and middorsally, granular-rugulose anteriorly; propleuron smooth; mesonotum weakly coriaceous; mesoscutum with short median posterior carina; notauli weakly scrobiculate; mesopleuron smooth and shinny, precoxal sulcus absent; epicnemial carina complete; propodeum smooth to weakly coriaceous dorsally, carinate-rugose basally, median carina complete. Legs:tarsal claws pectinate; hind coxa dorsally striate, same sculpture occasionally present on mid and fore coxa (Fig. 9). Wings (Fig. 15): dusky; R1/R2 0.29–0.33; R1/recurrent vein 0.38–0.4; 1CUa/1cu-a 1.7–2.5; basella/mediella 0.41–0.47.

Metasoma(Fig. 13): T1 length/width 1.19–1.25; T2 length/width 0.73–0.75; T3 length/width 0.48–051; T1 and T2 weakly rugose, apically smooth, raised triangular smooth area present; reminder metasomal terga smooth; OL/HBTL 0.21; HTS/HBTL 0.27–0.36.

Colour (Fig. 1): Black; mesopleuron, metapleuron and propodeum bright orange.

Male. Essentially as the female, but with slightly larger eyes; body length 10 mm; flagellum 59–63F.

##### Etymology.

The species is named in honour to Scott Shaw, for his contribution to the knowledge on this group.

**Figures 3–8.**
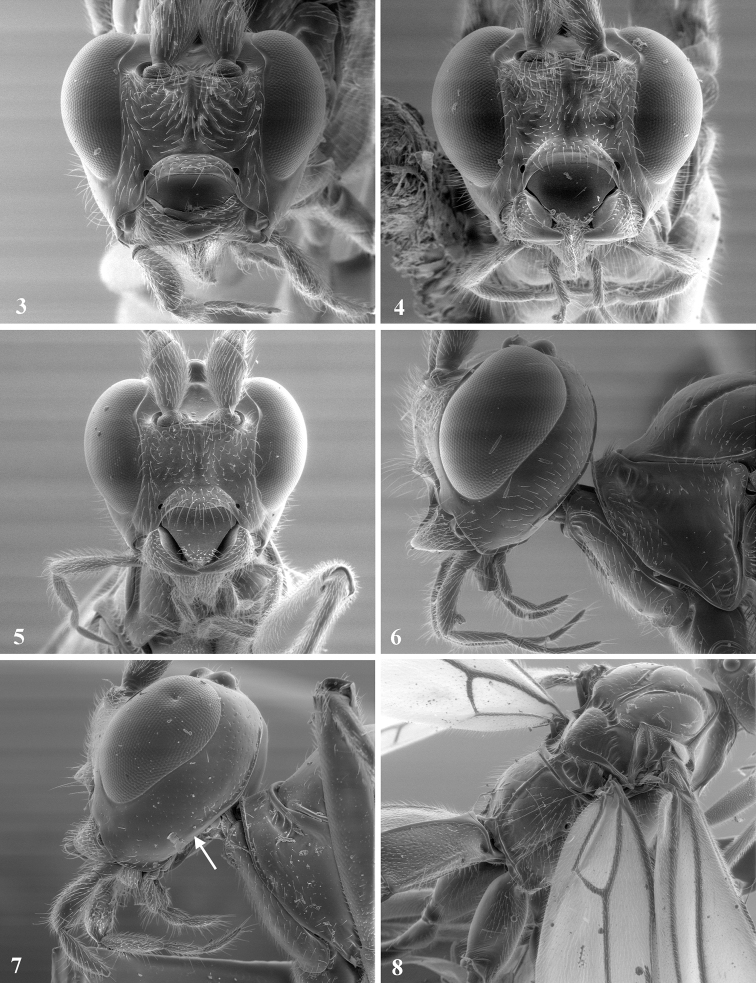
**3** *Aleiodes melanopterus* face **4** *Aleiodes shaworum* sp. n. face **5** *Aleiodes vassununga* sp. n. face **6** *Aleiodes shaworum* sp. n. head left **7** *Aleiodes melanopterus* head left arrow at end of occipital carina **8** *Aleiodes shaworum* sp. n. thorax dorsal.

**Figures 9–14.**
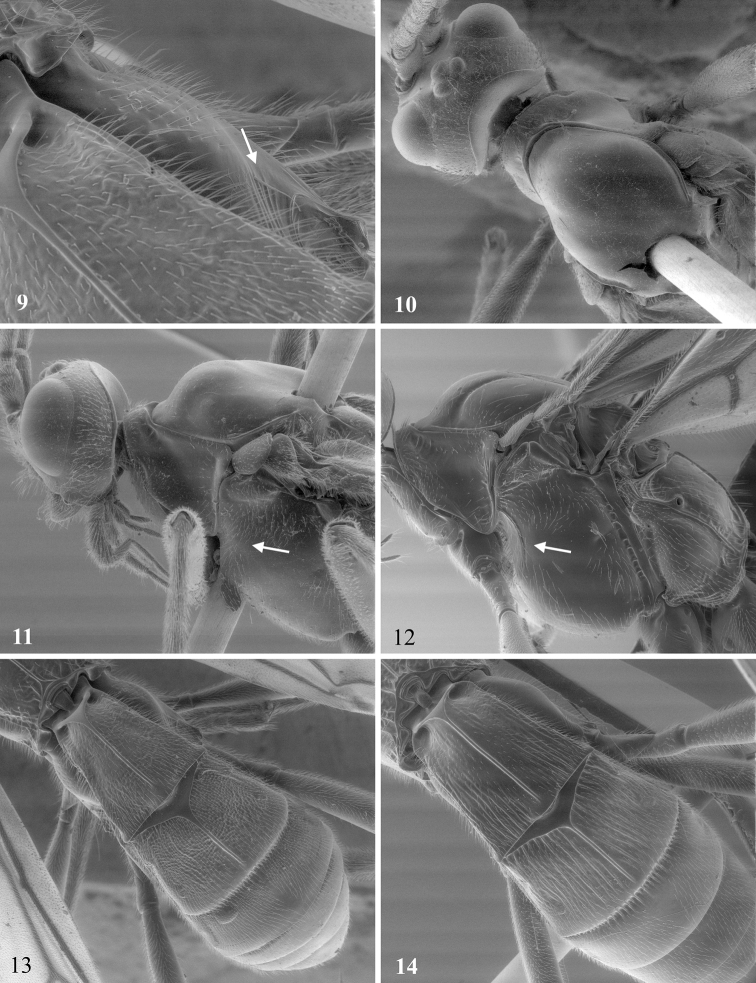
**9** *Aleiodes shaworum* sp. n.: part of first metasomal terga and hind coxa dorsal surface arrow at microsculpture striation on hind coxa. **10,11** *Aleiodes lucidus*: **10** head and mesoscutum dorsal **11** head and mesonotum left arrow indicating absence of epicnemial carina. **12** *Aleiodes shaworum* sp. n. mesopleuron left arrow at epicnemial carina. **13 14** Metasoma dorsal: **13** *Aleiodes shaworum* sp. n. **14** *Aleiodes vassununga* sp. n..

**Figure 15.**
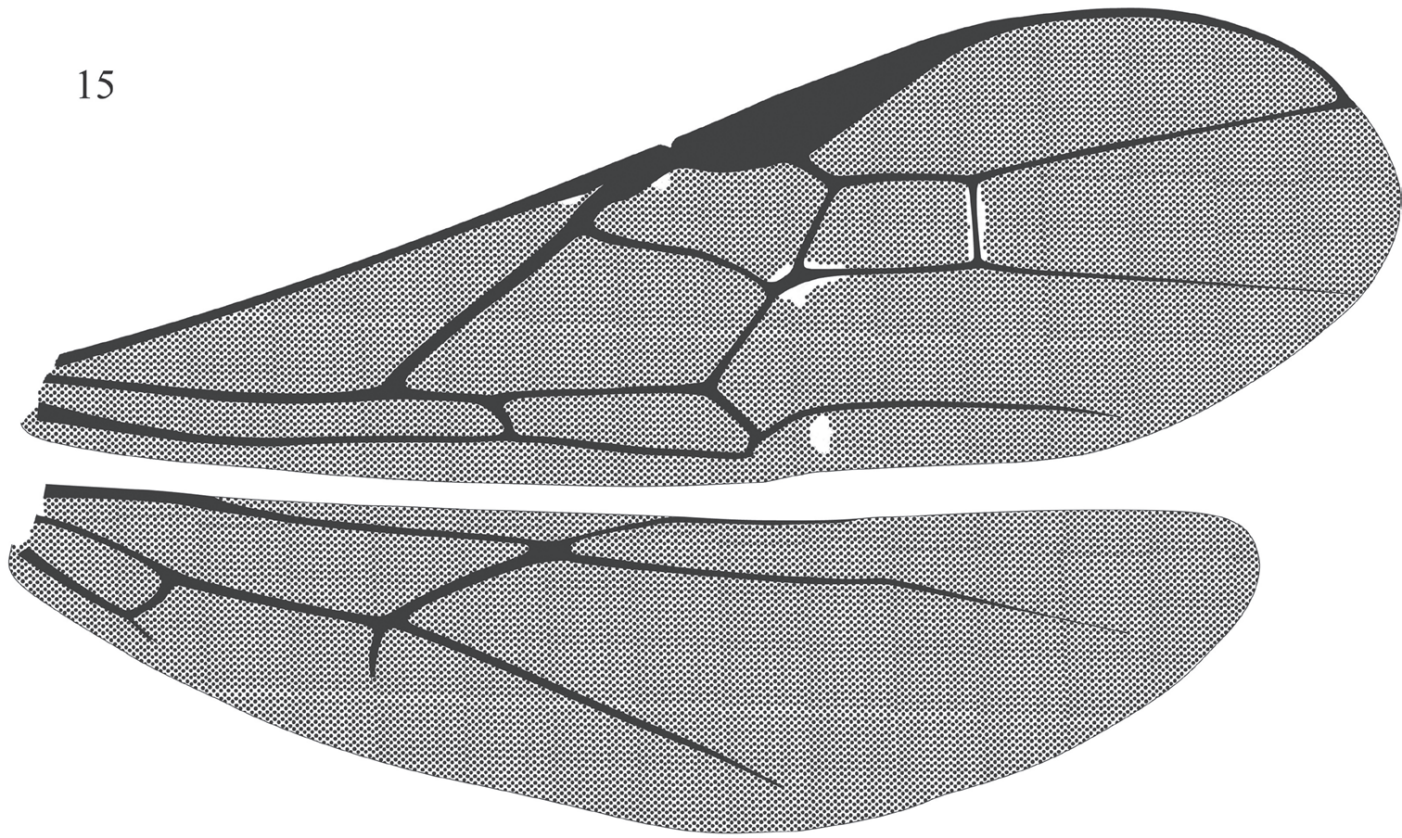
*Aleiodes shaworum* sp. n. right wings.

##### Distribution.

Brazil, State of São Paulo, Brazilian Atlantic Forest.

##### Comments.

Despite the superficial resemblance with *melanopterus*, this species have quite distinctive characters, including the lack of a strongly protruding clypeus, which has been considered one of the synapomorphies for the Neotropical *melanopterus* species-group clade (Shaw 1993; Fortier and Shaw 1999).

#### Aleiodes vassununga sp. n.

urn:lsid:zoobank.org:act:25462065-6F95-4324-9189-9D82350575B1

http://species-id.net/wiki/Aleiodes_vassununga

Figs 2 5 15

##### Material.

Type locality: Brazil, São Paulo, Santa Rita do Passa Quatro, Parque Estadual de Vassununga – Mata Praxedes, 21°40'56"S, 47°37'13"W, Semi-deciduous Atlantic forest.

Type specimens: Holotype, female, pinned. Original label: “Sta. Rita P. Quatro, SP, Brasil. Pq. Est. De Vassununga – Mata Praxedes – S21°40'56", W47°37'13" – 31.III.2006 – Armadilha Malaise – A.M.P. Dias col." DCBU / UFSCar, São Carlos.

Paratype (DCBU): 1 Male, Brazil, SP, Barueri, 6.XII.1966, K. Lenko col.

##### Diagnosis.

This species resembles the North American species *Aleiodes politiceps* and *melanopodus* Marsh and Shaw, 1999, both on body colour and the coarse sculpture on the metasoma, but it can be readily distinguished by the bright yellow spot on pterostigmal area of fore wing, as in *flavistigma*. In the key to North American species it will run to *Aleiodes melanopterus*, from which can be distinguished by the shorter ovipositor and smooth face sculpturing. It is also the only South American species without a black metasoma.

##### Description.

Female (Holotype). Body Length 10.8 mm, fore wing length 8.8 mm.

Head (Fig 5). Flagellum with 68 flagellomeres, F1 width 0.4 times its length, F15 0.8 times wider than long; malar space 0.3 times eye height, 0.8 times basal width of mandible; TW/EW 0.55; occipital carina not meeting hypostomal carina; oral opening width 1.26 times clypeo–antennal distance, OS/MS 2.5; OOD/OD 1.15; head entirely smooth; clypeus strongly protruding and margined by carina; maxillary palpus slightly swollen.

Mesosoma. Almost entirely smooth, pronotum sparsely rugose laterally; mesoscutum with short postero-median carina; notauli smooth, with some weak crenulae anteriorly; precoxal sulcus absent; epicnemial carina complete; propodeum smooth dorsally, carinate-rugose basally; median carina complete. Legs:tarsal claws pectinate; hind coxa dorsally smooth.Wings:dusky with yellow spot on pterostigmal area (Fig. 2); R1/R2 0.33; R1/recurrent vein 0.42; 1CUa/1cu–a 1.11; basella/mediella 0.39.

Metasoma(Fig. 14). T1 length/width 1.21; T2 length/width 0.76; T3 length/width 0.41; T1, T2 and basal half of T3 strongly costate; OL/HBTL 0.25; HTS/HBTL 0.25.

Colour (Fig. 2): Body entirely reddish brown, including scapus; antenna, ocelli, eyes, labial palpi, maxillary palpus segments 2–5 and ovipositor sheaths black; legs darkening apically from the tibia.

Male. Similar to female but face with some transverse rugositie; body length 11 mm; fore wing length 8.8 mm; flagellum with 63 flagellomeres.

##### Etymology.

The name of species refers to locality of collection of material for study.

##### Distribution.

Brazil, State of São Paulo.

## Supplementary Material

XML Treatment for Aleiodes flavistigma

XML Treatment for Aleiodes lucidus

XML Treatment for Aleiodes melanopterus

XML Treatment for Aleiodes shaworum

XML Treatment for Aleiodes vassununga
